# Dextrose Effects on Platelet Count and Volume: Implications for Regenerative Medicine

**DOI:** 10.7759/cureus.25081

**Published:** 2022-05-17

**Authors:** Theodore E Harrison, Jannice Bowler, K. Dean Reeves, Todd N Levins, An-Lin Cheng

**Affiliations:** 1 Regenerative Medicine, Private Practice, Sidney, CAN; 2 Pain Management, Private Practice, Victoria, CAN; 3 Rehabilitation Medicine, Private Practice, Kansas City, USA; 4 Integrative/Complementary Medicine, Private Practice, Victoria, CAN; 5 Biomedical and Health Informatics, University of Missouri Kansas City School of Medicine, Kansas City, USA

**Keywords:** platelet-rich plasma/prp, tissue regeneration, platelet activation, dextrose prolotherapy, blood platelets, prolotherapy

## Abstract

Platelet-rich plasma (PRP) and hypertonic dextrose solutions are commonly used injectates in regenerative medicine, sometimes used simultaneously. The effects of hypertonic dextrose on platelet lysis and activation have not been previously reported. We tested the effects of escalating dextrose concentration on cell counts and cell volume of platelets and red cells in PRP and whole blood (WB). A prompt partial reduction in platelet count occurred with all dextrose admixtures with either PRP or whole blood, consistent with partial lysis. After the first minute, platelet counts remained stable, suggesting a rapid accommodation of residual platelets to extreme (>2000 mOsm) hypertonicity. A 25% or higher dextrose concentration caused a significant increase in mean platelet volume (MPV), which suggests an early phase of platelet activation. Further investigation is warranted to confirm if platelet lysis or activation has occurred and whether additive clinical benefit may result from hypertonic dextrose injection alone or in combination with PRP.

## Introduction

In the 1950s, George Hackett, an American surgeon, discovered that he could permanently relieve joint and back pain in many patients by injecting the tendons and ligaments with a proliferative solution. His experiments in rabbits showed that this treatment, which he named prolotherapy, caused enlargement and strengthening of tendons. Histological study confirmed that new collagen was created during this process [1].

In the early decades, many different proliferant solutions were tried. By the 1990s, most practitioners had settled on high concentration dextrose as the safest and most effective. However, the mechanism of action remained obscure.

Few clinical studies were done in the twentieth century after Hackett’s work. However, in the 2000s, interest was rekindled and several successful clinical trials of prolotherapy for the treatment of lumbar pain [2], knee osteoarthritis [3], and lateral epicondylitis [4] were completed.

Tissue regeneration requires the participation of stem cells. Thus, high-concentration dextrose must somehow cause the migration, replication, and differentiation of stem cells. We theorized that platelets might be acting as the intermediary and that high concentration dextrose might cause the release of cytokines and growth factors from platelets, thus instigating the regenerative process, particularly the migration of stem cells to the area of high dextrose concentration.

Activation of platelets is always preceded by a rise in intracellular calcium [5]. Liu et al., in 2008, showed that high levels of glucose increased the activity of transient receptor potential canonical type 6 (TRPC6) channels in the plasma membrane, resulting in the influx of calcium ions into platelets [6]. Another study has shown that exposure of the marginal band of microtubules to calcium ions causes relaxation, expansion, and deformation of the marginal band, which, in turn, causes the shape change from discoid to globular resulting in an increase in mean platelet volume (MPV) [7].

Our hypothesis in this study was that the exposure of platelets to high concentrations of glucose causes effects on the marginal band of microtubules and intracellular milieu, resulting in an increase in MPV.

## Materials and methods

All participants had signed informed consent after the study details were explained and before samples were obtained. Only PRP samples with a hematocrit greater than 2% were used in this study in order to be able to include red blood cell (RBC) count and RBC mean corpuscular volume (MCV) for comparison purposes.

This study was performed in four stages, the first on PRP and the rest on whole blood (Table 1). All relative centrifugal forces (RCF, g forces) were calculated from the midpoint of the column of blood in the centrifuged syringe (Rmid, in cm) as described previously [8]. We chose to use MPV as a marker of platelet sensitization and platelet count as an indication of potential platelet lysis, both easily measured on standard hematology analyzers.

**Table 1:** Characteristics of stages one through four PRP = platelet rich plasma, WB = whole blood, MPV = mean platelet volume

Stages	PRP or WB volume (mL)	Dextrose volume	Original dextrose percent	Final dextrose percent	Time of measurement
Stage 1: Testing effect of dextrose on platelets in PRP n=29	0.5	0.5	0	0	Time 0 and 15 min
			5	2.5	
			12.5	6.1	
			25	12.5	
			50	25	
Stage 2: Testing effect of dextrose on platelets in WB n=20	2.0	2.0	50	25	Time 0 and 30 min
Stage 3: Evaluation of MPV and platelet count effects over time with 1 to 1 dilution of WB n=20	1.0	1.0	30	15	Time 0, 15 sec (immediately after placement in vortex mixer for 10 seconds), and every 2 min up to 30 min
			40	20	
			50	25	
Stage 4: Evaluation of MPV and platelet count effects over time with 1 to 5 dilution of WB n=20	0.2	1	25	20.8	Time 0, 15 sec and every 2 min up to 30 min
			50	41.7	

Stage 1: the effects of hypertonic dextrose on platelets in PRP over 15 minutes

In the first stage, 47 volunteers donated blood samples - one ethylene diamine tetra-acetic acid (EDTA) tube and a whole blood sample for PRP (which was anticoagulated with sodium citrate (NaCi, 3%) (Table 1). All samples were immediately placed into a test tube rocker. The EDTA samples were analyzed in triplicate for complete blood count (CBC) and the NaCi samples were analyzed in triplicate for CBC and then used to prepare PRP by various methods previously described [8]. All PRP samples were prepared by methods using centrifugation at 900 to 1000g. Each PRP sample was mixed for 5-10 seconds on a vortex mixer and five 0.5 mL aliquots were separated into test tubes.

To evaluate the effect of platelet exposure to escalating dextrose concentrations, equal amounts (0.5 ml) of 0%, 5%, 12.5%, 25%, and 50% dextrose in water were mixed with platelet samples to produce 0%, 2.5%, 6.25%, 12.5%, and 25% dextrose admixture concentrations, and the tubes were mixed for 15 minutes on a test tube rocker. Each mixture was analyzed for CBC in triplicate at 15 minutes. The platelet (PLT) count, RBC count, MCV, and MPV for each tube were averaged, and the mean PLT count, RBC count, MCV, and MPV were calculated across all PRP samples.

Stage 2: the effects of hypertonic dextrose on platelets in whole blood over 30 minutes at 1:1 dilution

After data collection for the first stage was completed, we noticed a significant increase in platelet volume in PRP platelets upon adding D50W. PRP platelets are not necessarily representative of all the platelets in the blood, neither is the PRP milieu the same as the WB milieu. Therefore, we elected to perform the second stage of testing on the effect of adding D50W to whole blood.

For the second stage, we chose a sample size of 30, based on the results of the first series, as described under “Analysis.” In this series, 20 volunteers donated blood samples (Table 1). Whole blood (1.8 cc) was drawn into a 3mL syringe, which was anticoagulated with 0.2 mL of 40% NaCi. The syringe of whole blood was mixed for five seconds with a vortex mixer and analyzed for CBC in triplicate. After analysis, the anticoagulated blood was added to 2 mL of 50% dextrose in a 5 cc syringe (resulting in a final dextrose concentration of about 25% (D25) and it was placed in a test tube rocker for 30 minutes. After 30 minutes, the content of the syringe of D25/WB was analyzed for CBC in triplicate. The PLT count, RBC count, MCV, and MPV for each syringe were averaged, and the mean PLT count, RBC count, MCV, and MPV were calculated for each sample before and after the addition of the dextrose.

Stage 3: the effects of dextrose on platelets in whole blood over time at 1:1 dilution

Since platelets within whole blood are routinely exposed to hypertonic dextrose during dextrose prolotherapy from the microtrauma of the injections, and the combination of PRP directly with hypertonic dextrose prior to injection is uncommon, we chose to study the effects of hypertonic dextrose in combination with WB in stages three and four. For each stage, 20 volunteers each donated 7-8 mL of ACD-A (Acid Citrate Dextrose Solution containing trisodium citrate (22.0 g/L), citric acid (8.0g/L), and dextrose (24.5 g/L)) anticoagulated blood (Table 1). Dextrose admixtures only in excess of 12.5% were used to determine a threshold percentage associated with MPV increase. In the third stage, 1 mL of blood was placed in a test tube. Then, while mixing the blood on a vortex mixer for 10 seconds, 1 mL of either 30% dextrose, 40% dextrose, or 50% dextrose was added to the test tube, resulting in final dextrose concentrations of 15%, 20%, and 25%, respectively. Immediately after mixing, the dextrose-blood sample was analyzed for CBC, and the analysis was repeated every two minutes for 30 minutes.

Stage 4: the effects of dextrose on platelets in whole blood over time at 1:5 dilution

The addition of hypertonic dextrose 1:1 with WB or PRP exposes platelets to higher concentrations than 25% for a few seconds during the initial mixing. In stage four, in order to evaluate the effect of hypertonic dextrose with a minimal initial spike in the concentration, and to test the upper limit of dextrose effects, we added only a small amount of blood to D25W or D50W. One mL of either D25W or D50W was placed in a test tube and 0.2mL of WB was added while mixing the sample on a vortex mixer for ten seconds. In these cases the blood was exposed to a dextrose concentration about 20% higher than the final concentration rather than 50% above the final concentration as in stage 3, resulting in final dextrose concentrations of 20.8% and 41.6%. The mixed samples were analyzed at the same intervals as in stage 3.

Analysis 

In the first stage for each dextrose dilution series, 30 samples were collected, as this is the proper sample size for a pilot study [9]. At the conclusion of each stage, including stage one, the sample size was evaluated for adequacy by employing the formula used for the determination of the sample size needed to estimate the mean of a continuous outcome variable in a single population. That formula is n = Z^2^ x SD^2^ /E^2^. In this equation, Z is the Z score, SD is the standard deviation, and E is the desired margin of error [10]. Our alpha was .05, with a corresponding Z score of 1.96, and our desired margin of error was 5 (percent). We were therefore solving for n = (1.96^2^ x SD^2^)/5^2^. The result shows that the required sample size for each stage was less than the actual number collected.

In Stages one, three, and four, where more than one concentration of dextrose was used, the effect of different dextrose concentrations was analyzed by comparing change scores between time 0 and each follow-up time (Stage one at 15 minutes, and Stages three and four at 15 seconds, and then every two minutes.) The change scores over each time period were compared by applying a Mann-Whitney U test since the data did not follow the normal distribution as determined by the Shapiro-Wilk test of normality. Bonferroni corrections were made to adjust the required alpha value to ≤0.01 rather than ≤0.05 due to 1 to 1 analysis of multiple groups (five) in Stage one and combined Stages three and four (five total).

## Results

Stage one results

**Figure 1:**
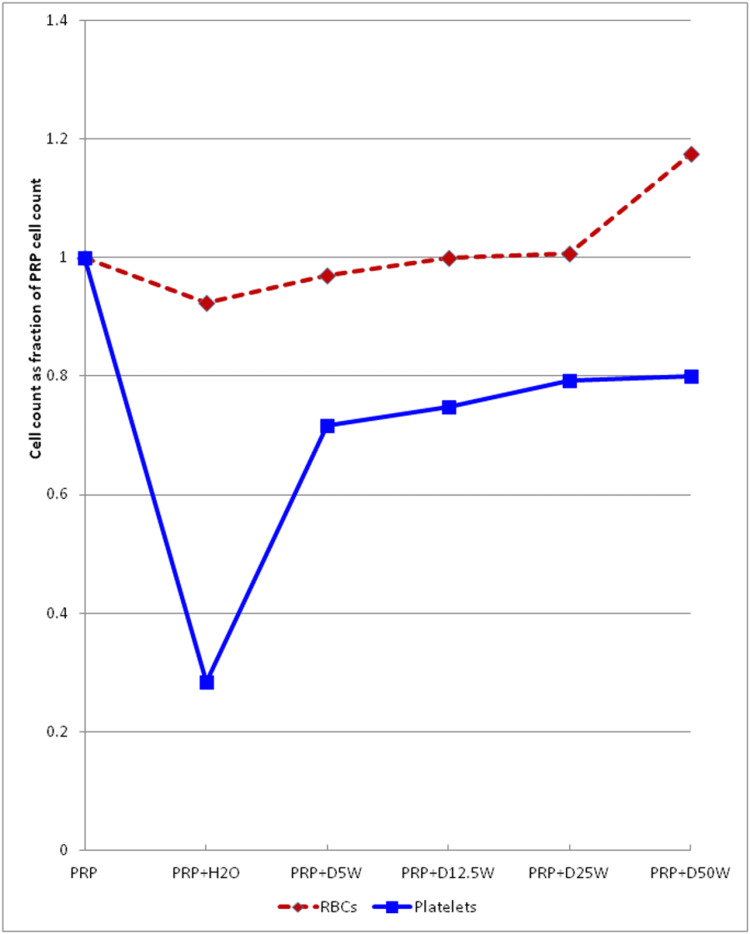
Comparison of cell numbers at different dextrose dilutions The effect of different dextrose concentrations on cell number. The number of cells in each dilution is expressed as a fraction of the original number before dilution.

Reduction of platelet count with all concentrations of hypertonic dextrose and an increase in MPV in PRP platelets at >12.5% dextrose concentration: PRP platelet counts rose from one to five times concentration compared to baseline whole blood, varying by the method (not depicted). Figure 1 shows that platelet counts, compared to that in baseline PRP and after volume correction of 1 to 1 dilution, dropped nearly 75% with dilution in water and by 20-30% with dilutions in different concentrations of dextrose over 15 minutes (volume corrected for 1 to 1 dilution).

**Figure 2:**
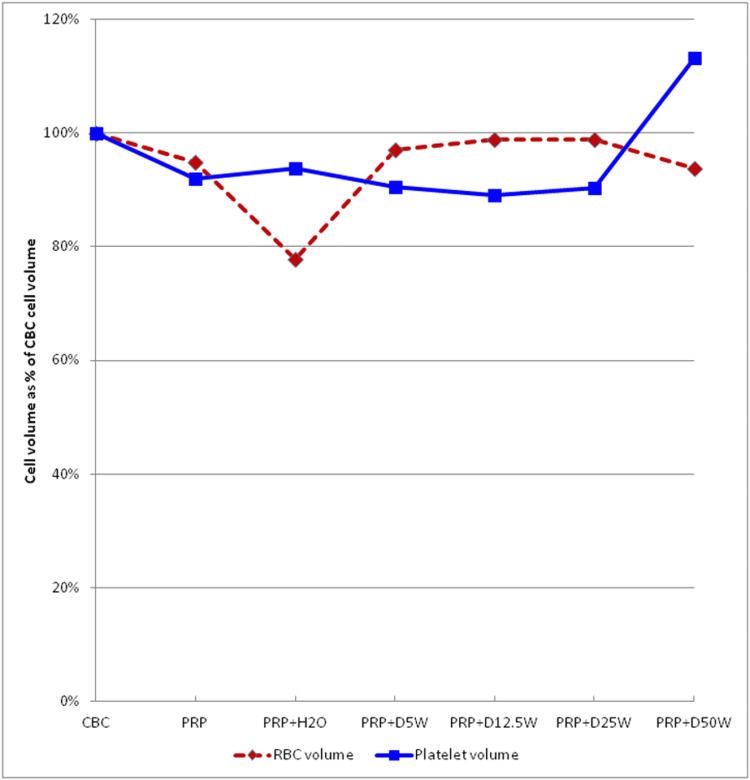
Comparison of cell volumes at different dextrose dilutions The effect of different dextrose concentrations on cell volume. The mean volume of cells in each dilution is expressed as a percentage of the original volume before dilution.

MPV decreased minimally during PRP production, did not change further with dilution in water or dextrose up to and including a diluted concentration of 12.5% (25% dextrose admixture with PRP), and increased by more than 20% upon dilution in 50% dextrose (Figure 2). In contrast, RBCs had no significant volume change at any dilution except with H2O.

Stage two results

Similar but less pronounced decreases in platelet count and increases in MPV within WB exposed to 50% dextrose (to make a 25% dextrose admixture) were observed. Table 2 compares cell counts and cell volume changes with whole blood dilution in dextrose 50% compared to values with PRP dilution in dextrose 50% from stage one. RBC count changes and MCV of red cells were not pronounced and were not our focus.

**Table 2:** RBC and PLT counts and cell volume before and after exposure to 50% dextrose for 15 minutes (PRP) and 30 minutes (WB) corrected for dilution effect SD = standard deviation, MD = mean difference between groups, SE = standard error of the mean difference, RBC = red blood cell, PLT = platelet, PRP = platelet rich plasma, WB = whole blood ^1 ^Corrected for dilution

WB or PRP	RBC Count Pre Exposure/SD	RBC Count Post^1^ Exposure/SD	RBC Change/SD/%	MCV Pre Exposure/SD	MCV Post Exposure/SD	MCV Change/ SD/ %	PLT Count Pre Exposure/SD	PLT Count Post^1^ Exposure/SD	PLT Change/ SD /%	MPV Pre Exposure/SD	MPV Post Exposure/SD	MPV Change/ SD/ %
WB (n = 23)	4.00/0.31	3.98/0.33	-.02/0.31/-0.5%	86.3/5.8	94.6/8.3	8.3/5.9 /+9.6%	310/73	286/96	-24/55 /-7.7%	10.1/0.5	11.8/0.6	1.7/0.8/+16.8%
PRP (n = 31)	0.66/0.45	0.74 0/46	+0.08/0.07/+12.1%	85.3/5.0	83.0/9.1	-2.3/6.3 /-2.7%	664/348	544/277	-118/109/-17.8%	9.2/0.8	11.6/0.7	2.4/1.0/+26%
MD (SE) P			.12(0.5) .004			10.6 (1.6) .001			94 (25) .001			0.7(0.2) .006

After addition of D50W to WB, the dilution-corrected platelet loss percentage was 7.7% (310 ± 73 versus 286 ± 96) in comparison to 17.8% (664 ± 348 versus 544 ± 277) platelet loss with PRP dilution in D50W. WB MPV increased by 16.8% (10.1 ± 0.5 to 11.8 ± 0.6) in comparison with a 26% increase in PRP MPV (9.2 ± 0.8 versus 11.6 ± 0.7). Although the mean differences in both platelet count reduction and MPV increase were significantly more with PRP, the changes in platelet count reduction within WB were nearly significant (310 ± 73 to 286 ± 96 (-7.7%); p = .06) and the increase in MPV was significant (10.1 ± 0.5 to 11.8 ± 0.6 (+16.8) p < .001).

Stage three results

**Figure 3:**
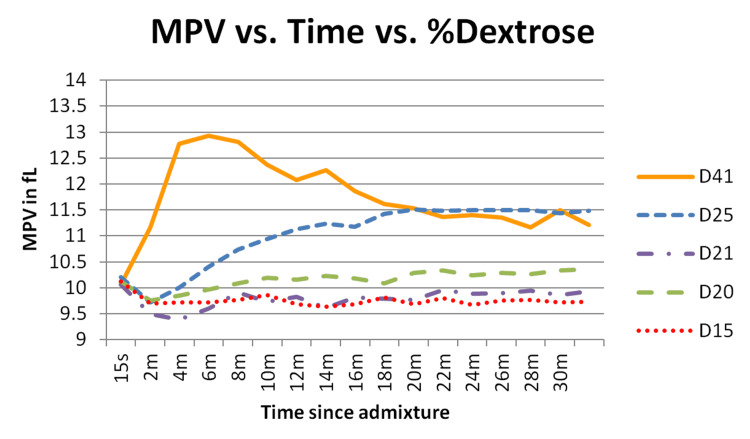
MPV vs. time at different dextrose concentrations Dextrose in different concentrations, when added to whole blood, causes an initial rapid drop in MPV followed by concentration-dependent recovery in concentrations greater than 20%. The legend shows the post-dilution dextrose concentrations. D15, D20, and D25 were performed at 1:1 dilution. D21 and D41 were performed at 1:5 dilution. MPV = mean platelet volume

A final concentration of 20% dextrose was required to see significant changes in MPV, but MPV changes were more pronounced with a 25% final concentration. Platelet losses stabilized after the initial drop. We noted an initial sharp decrease in MPV, however, the MPV rapidly recovered and, with a final dextrose concentration of 25%, significantly exceeded the MPV levels observed with final dextrose concentrations of 20% and 15% (Figure 3 and left side of Table 3; shaded boxes indicate p-values ≤ the Bonferroni adjusted alpha of .01). A sharp initial drop in PLT count was also observed, as seen in the initial period of 0-15 seconds and then remained stable thereafter (15 seconds to 30 minutes; left side of Table 4).

**Table 3:** Group by group comparison of mean difference (MD) in mean platelet volume (MPV) at each point in time over 30 minutes according to the final dilution MD = mean difference between groups, SE = standard error of the mean difference

		Stage 3 concentration comparisons			Stage 4 concentration comparisons and comparison with Stage 3 concentrations						
Time	Percent	25 v 20	25 v 15	20 v 15	42 v 25	42 v 20.8	42 v 20	42 v 15	25 v 20.8	20 v 20.8	20.8 v15
0 to 15s	MD (SE) p-value	0.1(0.1) .70	0.1(0.1) .69	0.1(0.2) .69	1.7(0.3) .001	1.8(0.3) .001	1.6(0.3) .001	1.6(0.2) .001	0.1(0.2) .52	0.2(0.2) .13	-0.1(0.2) .32
0 to 2m	MD (SE) p-value	0.1(0.2) .40	0.2(0.1) .17	0.1(0.2) .59	2.7(0.3) < .001	3.2(0.4) .001	2.8(0.4) .001	2.9(0.4) .001	0.5(0.2) .013	0.4(0.2) .05	-0.2(0.2) .15
0 to 4m	MD (SE) p-value	0.4(0.1) 0.031	0.6(0.1) .001	0.2(0.2) .08	2.7(0.3) < .001	3.3(0.3) .001	3.0(0.3) .001	3.2(0.3) .001	0.7(0.2) .001	0.3(0.2) .11	-0.1(0.2) .58
0 to 6m	MD (SE) p-value	0.5(0.2) .010	0.9(0.2) .001	0.4(0.1) .006	2.0(0.3) .001	2.7(0.3) .001	2.5(0.3) .001	2.9(0.3) .001	0.7(0.2) .002	0.2(0.2) .19	-0.2(0.2) .24
0 to 8m	MD (SE) p-value	0.7(0.1) .001	1.0(0.2) .001	0.3(0.2) .013	1.6(0.3) .001	2.7(0.3) .001	2.2(0.3) .001	2.6(0.3) .001	1.1(0.2) .001	0.4(0.2) .006	=0.1(0.2) .35
0 to 10m	MD (SE) p-value	0.9(0.2) .001	1.4(0.2) .001	0.5(0.2) .008	1.1(0.3) .002	2.1(0.3) .001	2.0(0.3) .001	2.5 (0.3) .001	1.1(0.2) .001	0.2(0.2) .20	-0.3(0.2) .06
0 to 12m	MD (SE) p-value	0.9(0.2) .001	1.5(0.2) .001	0.6(0.2) .002	1.1(0.2) .001	2.6(0.3) .001	2.0(0.2) .001	2.6(0.2) .001	1.5(0.2) .001	0.6(0.2) .008	0.0(0.2) .98
0 to 14m	MD (SE) p-value	0.9(0.2) .001	1.4(0.2) .001	0.5(0.2) .011	0.8(0.2) .002	2. (0.2) .001	1.7(0.2) .001	2.2(0.2) .001	1.2(0.2) .001	0.3(0.2) .11	-0.2(0.2) .32
0 to 16m	MD (SE) p-value	1.2(0.2) .001	1.5(0.2) .001	0.3(0.2) .09	0.3(0.2) .17	1.8(0.2) .001	1.6(0.2) .001	1.9(0.2) .001	1.5(0.2) .001	0.2(0.2) .13	0.0(0.2) 1.00
0 to 18m	MD (SE) p-value	1.1(0.2) .001	1.7(0.2) .001	0.6(0.2) .002	0.2(0.2) .46	1.8(0.2) .001	1.3(0.2) .001	1.9(0.2) .001	1.6(0.2) .001	0.5(0.2) .011	-0.1(0.2) .25
0 to 20m	MD (SE) p-value	1.1(0.2) .001	1.6(0.2) .001	0.5(0.2) .008	0.1(0.3) .89	1.5(0.3) .001	1.2(0.3) .001	1.7(0.3) .001	1.5(0.2) .001	0.4(0.2) .06	0.1(0.2) .60
0 to 22m	MD (SE) p-value	1.2(0.2) .001	1.7(0.2) .001	0.6(0.2) .002	0.0(0.2) .91	1.5(0.3) .001	1.2(0.3) .001	1.8(0.3) .001	1.5(0.2) .001	0.3(0.2) .18	0.3(0.2) .17
0 to 24m	MD (SE) p-value	1.1(0.2) .001	1.7(0.2) .001	0.6(0.2) .003	0.1(0.2) .91	1.5(0.2) .001	1.2 (0.2) .001	1.7(0.2) .001	1.5(0.2) .001	0.4(0.2) .05	0.2(0.2) .23
0 to 26m	MD (SE) p-value	1.1(0.2) .001	1.6(0.2) .001	0.5(0.2) .004	-0.1(0.2) .75	1.3 (0.3) .001	1.3 (0.2) .001	1.5(0.2) .001	1.4(0.2) .001	0.3(0.2) .08	0.2(0.2) .16
0 to 28m	MD (SE) p-value	1.0(0.2) .001	1.6(0.2) .001	0.6(0.2) .001	0.3(0.2) .44	1.7(0.2) .001	1.0 (0.2) .001	1.9(0.2) .001	1.4(0.2) .001	0.4(0.2) .05	0.2(0.2) .19
0 to 30m	MD (SE) p-value	1.1(0.2) .001	1.7(0.2) .001	0.6(0.2) .001	-0.1(0.2) .25	1.4 (0.2) .001	1.1(0.2) .001	1.6(0.2) .001	1.5(0.2) .001	0.4(0.2) .04	0.3(0.2) .07

**Table 4:** Platelet counts by group at times 0 seconds, 15 seconds, and 30 minutes, along with immediate percentage drop in volume-corrected platelet accounts (0 to 15 seconds) and drop in platelet count over 30 minutes SD = standard deviation, plt = platelets ^1^ Percentage of time 0 platelet count)

	Stage 3: One to one dilutions			Stage 4: One to five dilutions	
	15% (30à15)	20% (40à20)	25% (50à25)	20.8% (25à20.8)	41.7% (50à41.7)
Time 0 plt count	289 ± 46	289 ± 46	286 ± 39	265 ± 59	266 ± 60
15-sec plt count Mean ± SD	232 ± 58	221 ± 66	215 ± 35	179 ± 55	154 ± 69
30-min plt count Mean ±SD	208 ± 34	209 ± 57	213 ± 48	142 ± 47	159 ± 52
0 to 15-sec Mean ± SD (%)^1^	-57 ± 57 (20%)	-69 ± 49 (24%)	-71 ± 39 (25%)	-86 ± 59 (32%)	112 ± 78 (42%)
15-sec to 30-min Mean ± SD (%)^1^	-15 ± 49 (9%)	-11 ± 67 (4%)	-3 ± 44 (1%)	-37 ± 63 (14%)	+5 ± 68 (2%)

Stage four results

**Figure 4:**
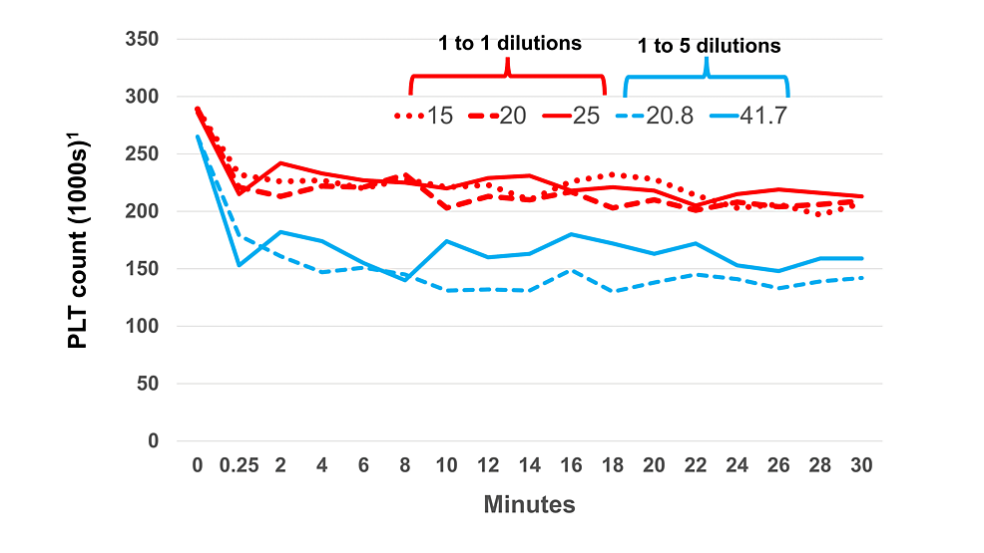
Dextrose effect on platelet counts at different concentrations and dilutions When whole blood is added to dextrose in a 1:1 ratio, the platelet count decreases by about 25%. However, when whole blood is added at a 1:5 ratio, the decrease is much larger - approximately 50%. ^1 ^(corrected for volume)

Table 4 lists the changes in platelet count for every dilution in hypertonic dextrose. We observed a dose-dependent relationship between the immediate drop in PLT count in the 1:1 dilutions versus the 1:5 dilutions. By combining 1:1 dilutions as one group for comparison with the 1:5 dilutions, the 1:1 group showed a smaller immediate drop in PLT count than the 1:5 group 66±48 thousand (23%) versus 99±69 thousand (37%, p=0.014) in the 1:5 group. Platelet counts stabilized for each dextrose percentage after the initial drop at the first measurement point (Figure 4).

Forty-one percent dextrose elevated MPV faster and more substantially than 25% or 21%. The MPV results are depicted in Figure 3. With the addition of 50% dextrose, there was no detectable immediate initial decrease in MPV as was observed in all other dilutions. With 25% dextrose (final diluted dextrose concentration 20.8%), the MPV change was comparable to the 20% dextrose MPV change in the 1:1 dilutions (Figure 3). Although MPV changes were initially more significant with an admixture concentration of 41% than with 25%, the difference in MPV between 41% and 25% was no longer significant after 16 minutes (right side of Table 3). Also of interest was that 25% dextrose raised MPV more effectively than 20.8%.

## Discussion

This in-vitro study partially confirmed our hypothesis. It showed potential partial platelet lysis by dextrose admixture, a rapid accommodation of platelets to extreme hypertonicity, and a significant rise in MPV in response to > 25% concentrations of hypertonic dextrose. The initial rise was the most with 41.6% dextrose exposure, but the MPV increase approximated that of 25% dextrose exposure after about 20 minutes.

Platelet concentration is affected by glucose. We noted that the PLT count decreases at all dextrose dilutions. The dramatic drop in platelet count in the H2O (0%) dilution of the PRP series may be due to osmotic lysis. Alternatively, it might be an artifact caused by platelet clumping, but the lack of change in the MPV at this dilution argues against that. This finding implies that some platelets are very sensitive to low osmolarity.

The 20-30% drop in the PLT count across all dextrose 1:1 dilutions, even at D5W (which is hypo-osmolar at 252 mOsm), may indicate a particular non-osmotic dextrose effect, as the PLT and MPV both remain constant in the three increasing dextrose concentrations from D5W to D25W. In fact, there is a slight trend toward increasing PLT concentration with increasing osmolarity.

The decrease in PLT between 1:1 dilutions and 1:5 dilutions implies a lytic effect that is dependent on both the initial and final concentrations of dextrose. If it were only dependent on the initial concentration then we would expect to see differences in PLT decrease between the 1:1 concentrations. But we do not. If the lytic effect were dependent only on the final concentration of dextrose then we would not expect to see much difference between the 20% 1:1 dilution and the 20.8% 1:5 dilution. Yet we do.

If the platelet loss is due to platelet lysis then a partial lysate is being formed with consequent release of the cytokines and growth factors into the extracellular environment. Some studies have shown that platelet lysate is almost as potent as PRP as a proliferant solution [11]. PRP itself has been shown to be an effective prolotherapy proliferant solution [12-14].

Inactive platelets circulate in a discoid shape reinforced by several internal structures. During activation, they change to a more globular or ameboid shape, which causes an increase in volume. That increase in volume necessitates an increase in surface area, which comes from both extrusion of the open canalicular system (OCS) and the addition of membrane from exocytosed granules. It remains to be determined whether the MPV increase caused by hypertonic dextrose involves one or both of these mechanisms, but if the latter is the case then the increase in MPV would be indicative of degranulation.

This study found that exposure of platelets within PRP or whole blood to a high concentration of glucose resulted in an increase in MPV within 15 minutes in dextrose concentrations of 25% and 41.6%.

An increase in platelet MPV may result from the expansion of the circumferential microtubule coil, which is dependent on calcium influx. Liu et al. showed that glucose mediates calcium influx through the TRPC6 channels of platelets [6]. Our hypothesis was that dextrose would cause relaxation of the microtubular coil, resulting in increased MPV and sensitization and/or activation of platelets. However, it appears from our results that this is only partially the case. No concentration less than D25W produced increased MPV in our tests. Our Stage 1 results, given that we did not test exposure to dextrose concentrations between 12.5% and 25%, indicated that there was a likely threshold within that range of dextrose concentration that results in an increase in MPV. Further testing in Stages 3 and 4 indicates that 20-25% dextrose seems to be the threshold at which this occurs, but it remains unclear why.

We also observed that MPV decreases by about 9% after centrifugation. It is unclear whether this decrease in MPV results from larger, higher-density platelets becoming trapped in the RBC layer of the centrifugate. This observation may be important for the clinician, as it potentially implies that PRP platelets are a smaller, less dense subset of WB platelets.

In a previous study, we showed that PRP preparation by manual methods need not be expensive [8]. If dextrose sensitizes platelets in either tissue or PRP, and thus causes them to activate more readily, or if PRP with features of a partial lysate is produced, regeneration may be enhanced and fewer treatments required. Therefore, it is possible that a combination of PRP and high concentration dextrose may prove to be more cost-effective than PRP or dextrose alone.

Our study has several weaknesses. First is the fact that we used PRP made from a number of different methods. This may have caused inconsistent results. Second, we were unable to do biochemical analysis on any of our samples to more accurately determine whether platelet activation had occurred. We would have liked to have measured P-selectin, platelet factor 4, monocyte-platelet-aggregates, or other markers of platelet activation to get a better idea of the extent or existence of alpha granule degranulation, but that was beyond the scope of this study. Third, we were unable to confirm by electron microscopy or other means that the increase in MPV of platelets exposed to dextrose was due to an effect on the microtubular coils.

## Conclusions

Admixture of WB or PRP with 25% dextrose increased MPV, a signal of the onset of platelet activation, although this study did not confirm progression to aggregation or degranulation. Hypertonic dextrose admixture resulted in platelet loss, potentially representing a lytic effect. Either partial platelet activation or platelet lysis may induce tissue regeneration after platelet injection. It is unclear what clinical effects these changes may cause. Further studies are indicated with more precise activation or lysis measurements and evaluating differential clinical effects of hypertonic dextrose admixtures with WB or PRP.

Dextrose prolotherapy is a simple, inexpensive, regenerative therapeutic technique, with rapidly expanding and supportive clinical studies. This study proposes a physiological mechanism, which, if confirmed, may contribute to our understanding of a portion of the regenerative mechanism of prolotherapy.
